# The Radical Scavenging Activities and Anti-Wrinkle Effects of Soymilk Fractions Fermented with *Lacticaseibacillus paracasei* MK1 and Their Derived Peptides

**DOI:** 10.3390/antiox12071392

**Published:** 2023-07-06

**Authors:** Sulhee Lee, Sang-Pil Choi, Huijin Jeong, Won Kyu Yu, Sang Won Kim, Young-Seo Park

**Affiliations:** 1 Kimchi Functionality Research Group, World Institute of Kimchi, Gwangju 61755, Republic of Korea; slee@wikim.re.kr (S.L.); spchoi@wikim.re.kr (S.-P.C.); 2 Department of Food Science and Biotechnology, Gachon University, Seongnam 13120, Republic of Korea; gmlwls2218@gachon.ac.kr; 3 Yonsei University Dairy, Asan 31419, Republic of Korea; wonkyu@yonseidairy.com (W.K.Y.); ksw@yonseidairy.com (S.W.K.)

**Keywords:** lactic acid bacteria, radical scavenging activity, anti-wrinkle, anti-aging, soybean milk, fermentation, peptide

## Abstract

Soybean-derived peptides exert several beneficial effects in various experimental models. However, only a few studies have focused on the radical scavenging and anti-wrinkle effects of soymilk-derived peptides produced via different processes, such as fermentation, enzymatic treatment, and ultrafiltration. Therefore, in this study, we investigated the radical scavenging and antiwrinkle effects of soymilk fractions produced using these processes. We found that 50SFMKUF5, a 5 kDa ultrafiltration fraction fermented with *Lacticaseibacillus paracasei* MK1 after flavourzyme treatment, exhibited the highest radical scavenging activity using the 2,2-diphenyl-1-picrylhydrazyl radical scavenging assay as well as potent anti-wrinkle effects assessed by type 1 procollagen production and tumor necrosis factor-α production in ultraviolet B (UVB)-treated human dermal fibroblasts and HaCaT keratinocytes. To identify potential bioactive peptides, candidate peptides were synthesized, and their anti-wrinkle effects were assessed. APEFLKEAFGVN (APE), palmitoyl-APE, and QIVTVEGGLSVISPK peptides were synthesized and used to treat UVB-irradiated fibroblasts, HaCaT keratinocytes, and α-melanocyte-stimulating hormone-induced B16F1 melanoma cells. Among these peptides, Pal-APE exerted the strongest effect. Our results highlight the potential of soymilk peptides as anti-aging substances.

## 1. Introduction

Increasing demand for plant-based protein sources and non-dairy milk alternatives has brought soybean (*Glycine max*) and soymilk to the forefront of nutritional research. Soybeans are valuable protein sources that have been extensively studied for their nutritional benefits. They are considered viable alternatives to animal products, such as milk and meat, and are one of the most consumed protein sources worldwide [1]. Soymilk, a soybean product, is a popular non-dairy milk alternative owing to its low cost, improved functional and nutritional properties, and potential health benefits. Soymilk is a good nutritional medium for the growth and proliferation of microorganisms, indicating its potential as a carrier for probiotics and other bioactive compounds [2].

Soybean-derived peptides are protein fragments produced from soybean proteins via different processes, such as hydrolysis, fermentation, food processing, and gastrointestinal digestion. These methods enable the production of many small peptides with radical scavenging properties from soy protein [3]. Some of these peptides exert numerous beneficial effects, such as antidiabetic, anticancer, hypotensive, anti-inflammatory, and radical scavenging effects, in various experimental models. Soybean peptides have an average molecular weight of approximately 3–10 kDa and high glutamic acid content [4]. Enzymes, bacteria, and types of soy protein affect the composition and production of peptides [5]. The use of microorganisms to ferment substances is a cost-effective approach for the production of bioactive peptides that is extensively employed in the dairy industry to improve the functionality of milk products and byproducts. Several fermented dairy products have functional health benefits linked to bioactive peptides. The majority of these products are obtained using lactic acid bacteria [6]. Furthermore, fermented soybean meal, soy foods, and soymilk exhibit high potency in combating oxidative stress [7,8,9].

Several studies have highlighted the association between the radical scavenging capacity and the anti-aging effects of different substances [10,11]. Aging is a natural process affecting all living organisms. Skin ages more rapidly than other parts of the body because of direct exposure to environmental factors, such as chemicals and solar ultraviolet (UV) radiation [12]. Excessive exposure to UVB radiation (290–320 nm) causes photoaging, which is characterized by dry and rough skin with wrinkles, irregular pigmentation, and reduced elasticity [13]. Exposure of the skin to UV radiation for an extended period stimulates the production of reactive oxygen species (ROS) in the epidermis, leading to oxidative stress [14]. ROS can activate keratinocytes to produce pro-inflammatory cytokines, such as interleukin (IL)-1, -6, and -8, and tumor necrosis factor (TNF)-α [15]. Subsequently, these cytokines induce the expression of matrix metalloproteinases (MMPs) in dermal fibroblasts [16]. MMPs degrade collagen and other proteins in the connective tissue. Moreover, increased MMP activity combined with decreased procollagen production impairs the skin structure, ultimately leading to wrinkle formation during skin photoaging [17].

In this study, we aimed to investigate the radical scavenging and anti-wrinkle effects of soymilk-derived peptides produced via different processes, namely fermentation, enzymatic treatment, and ultrafiltration (UF). The radical scavenging activities of soymilk-derived peptides were measured using the 2,2-diphenyl-1-picrylhydrazyl (DPPH) radical scavenging assay, and their anti-wrinkle effects were evaluated based on type 1 procollagen synthesis and TNF-α production in UVB-radiation-exposed human dermal fibroblasts and HaCaT keratinocytes. Furthermore, liquid chromatography–tandem mass spectrometry (LC-MS/MS) analysis was used to identify their potential bioactive peptides. Finally, candidate peptides were synthesized based on the analysis results, and their anti-wrinkle effects were evaluated.

## 2. Materials and Methods

### 2.1. Lactic Acid Bacteria and Fermentation of Soymilk

The strain and optimal conditions used for soymilk fermentation were as previously reported [18]. *Lacticaseibacillus paracasei* MK1 was isolated from aged kimchi and soymilk was obtained from Yonsei University Milk (Seoul, Republic of Korea). Sterilized soymilk was inoculated with *L. paracasei* MK1 to a viable count of 9.0 log CFU/mL and fermented at 30 °C for 18 h for subsequent use in this study.

### 2.2. Soymilk Sample Preparation

#### 2.2.1. Commercial Protease Sample

Commercial protease-treated soymilk was prepared using protamex (Novozyme, Bagsværd, Denmark), a blend of microbial endo-proteases from *Bacillus subtilis*, or flavourzyme (Novozyme), a high-quality blend of endo- and exo-peptidases from *Aspergillus oryzae*, to compare *L. paracasei* MK1-fermented and commercial protease-treated soymilk. Protamex or flavourzyme (2 g of enzyme per kg of protein) was added to sterilized soymilk preheated to 50 °C, shaken for 4 h at the same temperature, and inactivated by heating at 80 °C for 10 min.

#### 2.2.2. Fractionation of Soymilk Samples

UF was performed to fractionate the MK1-fermented and commercial protease-treated soymilk. MK1-fermented or protease-treated soymilk was centrifuged at 12,000× *g* for 20 min, and the supernatant was filtered via membrane filtration using a nitrocellulose filter membrane (Sigma, St. Louis, MO, USA) with 0.22 µm pore size, and the filtrate was used for UF. The Labscale TFF system (Millipore Corporation, Bedford, MA, USA) was used as a UF device, and cartridges were performed using BIOMAX 30 K and 5 K polyethersulfone (50 cm^2^; Pellicon XL filter, Millipore Corporation). All soymilk samples prepared using this method are listed in Table 1.

### 2.3. Determination of the Radical Scavenging Activities of Soymilk Samples Using the DPPH Radical Assay

The radical scavenging activities of soymilk samples were determined using the DPPH radical scavenging assay as described by Liu et al. [19], with slight modifications. Briefly, the soymilk samples were mixed with 500 μM DPPH (Sigma) dissolved in ethanol in a ratio of 1:1 and kept in the dark at 20 °C for 20 min. Absorbance was measured at 540 nm using a microplate reader (Epoch, BioTek Instruments, Inc., Winooski, VT, USA). Ascorbic acid (Sigma) at a concentration of 1 mM was used as a positive control. The DPPH radical scavenging activity was calculated as follows:

DPPH radical scavenging activity (%) = (A_blank_ − A_sample_)/A_blank_ × 100 (1)

where A_blank_ is the absorbance of the blank, and A_sample_ is the absorbance of the sample.

### 2.4. Treatment of Cell Lines with Soymilk Fractions

#### 2.4.1. Cells and Reagents

Normal human dermal fibroblast (fibroblast) and non-tumorigenic human keratinocyte (HaCaT) cell lines from the American Type Culture Collection (Manassas, VA, USA) and melanoma murine B16F1 cell line from the Korean Cell Line Bank (Seoul, Republic of Korea) were cultured in the Dulbecco’s modified Eagle’s medium (DMEM; Welgene, Daegu, Republic of Korea) supplemented with 10% fetal bovine serum (FBS; Welgene) and 1% penicillin/streptomycin (Welgene). Cells were grown at 37 °C with 5% CO_2_ in an incubator.

#### 2.4.2. Cell Viability Assay

Cell viability was assessed using 3-(4,5-dimethylthiazol-2-yl)-2,5-diphenyltetrazolium bromide (MTT; Sigma) as described by Kumar et al. [20]. Fibroblasts, HaCaT cells (7 × 10^3^ cells/well), and B16F1 melanoma cells (5 × 10^3^ cells/well) were treated with various concentrations of soymilk samples for 24 h. After adding 100 μL/well of MTT solution, the cells were incubated at 37 °C. After 90 min, the MTT solution was removed, 100 μL/well of dimethyl sulfoxide (Sigma) was added, and cells were incubated at 20 °C. The plates were gently shaken, and the absorbance at 570 nm was determined using a microplate reader (Perkin Elmer, Waltham, MA, USA).

#### 2.4.3. UVB Exposure of Fibroblasts and HaCaT Cells

Human fibroblasts were seeded in a 6-well plate at a density of 2 × 10^5^ cells/well. After 24 h of incubation, the medium was replaced with a serum-free medium for starvation. The UVB irradiation method consisted of replacing phosphate-buffered saline (Amresco LLC, Solon, OH, USA) after washing the cells, which were then exposed to UVB irradiation at 312 nm and 25 mJ/cm^2^ using a VLX-3 W research radiometer (Vilber Loumet, Collégien, France). Then, cells were treated with various concentrations of soymilk in DMEM without FBS for 48 h. Then, the procollagen content in the culture supernatant was measured using the procollagen type-I C-Peptide EIA kit (Takara Bio, Inc., Shiga, Japan). Ascorbic acid (50 μM; Sigma) was used as a positive control, cells not treated with UV light were used as negative controls, and untreated soymilk samples were used as controls.

HaCaT keratinocytes were seeded in a 12-well plate at a density of 1 × 10^5^ cells/well and incubated for 24 h. Then, HaCaT keratinocytes were treated with various concentrations of soymilk samples under UVB irradiation (at 312 nm and 12.5 mJ/cm^2^) and incubated for 24 h. After incubation, the culture supernatant was analyzed for TNF-α secreted by the soymilk samples using the TNF alpha human enzyme-linked immunosorbent assay (ELISA) kit (#KHC3012; Invitrogen, Carlsbad, CA, USA). We used 1 μM/mL of 50 μM dexamethasone (Sigma) as a positive control, and cells not treated with UV as negative controls.

#### 2.4.4. Melanin Content of B16F1 Cells

Melanin production in cells was measured using a modified version of the method described by Tsuboi et al. [21]. B16F1 melanoma cells were treated with samples derived from the soymilk fractions in media for three days. After incubation, the cell pellets were harvested and dissolved in 1 mL of 1 N NaOH at 100 °C for 30 min. The dissolved samples were centrifuged, and the absorbance of the supernatant was measured at 400 nm.

### 2.5. Chemical Parameters

The saccharide, carbohydrate, crude protein, crude lipid, and solid contents of the original soymilk were analyzed by the Korea Standards Test and Analysis Institute (Gyeonggi, Republic of Korea). Crude protein and lipid contents of the fermented soymilk fractions were determined by the Korea Food Research Institute (Wanju, Republic of Korea). Free amino acids were analyzed by the Korea Basic Science Institute (Daejeon, Republic of Korea). The high-performance liquid chromatography conditions are presented in Table S1. Analysis of peptides in soymilk samples was performed by the National Instrumentation Center for Environmental Management (Seoul, Republic of Korea), and the analysis conditions are presented in Table S2. Total nitrogen content was analyzed following the macro-Kjeldahl method [22] using the Kjeltec 8100 distilling and 2508 digestion units (Foss, Eden Prairie, MN, USA). Trichloroacetic acid (TCA)-soluble nitrogen content was determined as described by Rowland [23]. Briefly, 10 mL of the sample and the same amount of 24% (*w/v*) TCA solution were mixed well, incubated at room temperature for 30 min, and centrifuged at 8000 rpm for 15 min to measure the TCA-soluble nitrogen content in the supernatant. The amount of reducing sugar released was determined following the Somogyi–Nelson method [24], with slight modifications [25].

### 2.6. Statistical Analyses

Statistical analyses were conducted using the GraphPad 6.01 software (GraphPad Software Inc., San Diego, CA, USA). Data were analyzed using analysis of variance, followed by Dunnett’s test for pairwise comparisons. Data are presented as the mean ± standard deviation. Statistical significance was set at * *p* < 0.05, ** *p* < 0.01, *** *p* < 0.001, and **** *p* < 0.0001 for all tests.

## 3. Results and Discussion

### 3.1. DPPH Radical Scavenging Activities of Soymilk Fractions

**Figure 1:**
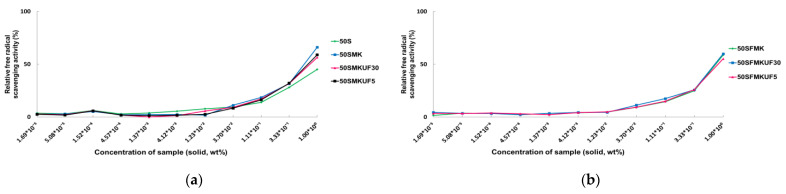
2,2-diphenyl-1-picrylhydrazyl (DPPH) radical scavenging activities of various soymilk fractions. (**a**) 50S, 50SMK, 50SMKUF30, and 50SMKUF5. (**b**) 50SFMK, 50SFMKUF30, and 50SFMKUF5.

Fermentation using lactic acid bacteria produces bioactive antioxidants, which maintain food stability and are beneficial to humans [5]. In this study, after lactic acid bacterial fermentation and/or enzymatic treatment of soymilk, the radical scavenging activities of various fractions were determined via UF. DPPH radical scavenging activities were determined to measure the radical scavenging activities of the soymilk fractions. The radical scavenging activities of the soymilk fractions (Table 1) were measured, and ascorbic acid was used as a positive control. DPPH radical scavenging activity increased in a dose-dependent manner (Figure S1), and 50SMK with 50% soymilk fermented with *L. paracasei* MK1 exhibited the highest radical scavenging activity, with 66.11% scavenging activity (Figure 1). By contrast, the fractions without fermentation or enzyme treatment (50S) exhibited the lowest radical scavenging activity of 45.11%, with 50–60% DPPH radical scavenging activity.

Next, we determined the half-maximal inhibitory concentration (IC_50_), the point at which the radical scavenging activity is reduced by half, of the samples (Table 2). Except for 50S and 50SMK, no significant differences were observed among the other fractions. The 50S fraction had an IC_50_ of 1.24%, which was much higher than that of the other fractions, and 50SMK had an IC_50_ of 0.67%, which was lower than that of the other fractions. The DPPH radical scavenging activities and IC_50_ values of different fractions were similar, but the radical scavenging activity was the highest for 50SMK.

The fermentation of soymilk by lactic acid bacteria produces isoflavones and peptides [26,27,28,29]. Soymilk fermented with *L. paracasei* KUMBB005 reduces the DPPH radical scavenging activity by 27% [30], whereas that fermented with *L. paracasei* CD4 improves the free radical scavenging activity by 67% [31]. Soymilk fermented with *L. paracasei* MK1 exhibited high radical scavenging activity, possibly due to the peptides or isoflavones present in fermented soymilk.

### 3.2. Cytotoxicities of Soymilk Fractions in Cell Lines

The cytotoxicities of various soymilk fractions with radical scavenging activity were determined using a skin cell line. MTT reagent was used to measure the cytotoxicities of the soymilk fractions. The MTT assay measures the absorbance of formazan (purple) obtained after the reduction of MTT (yellow) by succinate dehydrogenase in the mitochondria to reflect the concentration of active cells [32].

#### 3.2.1. Cytotoxicities of Soymilk Fractions in Fibroblasts

**Figure 2:**
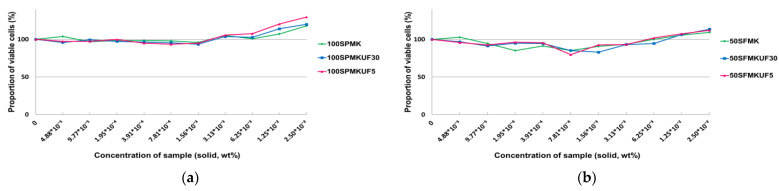
Effects of various soymilk fractions on the viability of human dermal fibroblasts. (**a**) 100SPMK, 100SPMKUF30, and 100SPMKUF5. (**b**) 50SFMK, 50SFMKUF30, and 50SFMKUF5.

Cytotoxicity was evaluated by treating cells with various concentrations of soymilk fractions and comparing them to a control group with untreated cells (Figure S2). In particular, the highest growth rate of 129.67% was observed when fibroblasts were treated with ultrafiltered 100SPMKUF5, which was obtained by fermenting protamex-treated 100% soymilk with *L. paracasei* MK1 (Figure 2). This growth rate was observed at a concentration of 2.50 × 10^2^. No significant differences were observed among all treated soymilk fractions. However, when fibroblast cells were treated with a concentration ≥ 1.25 × 10^2^, the survival rate was higher than that of the control. Glycitin, a soybean isoflavone, exhibited non-cytotoxicity and increased cell proliferation in dermal fibroblasts in a dose-dependent manner [33]. Treating fibroblasts with soy peptides results in a non-toxic outcome similar to that in the positive control, AA2G (vitamin C derivative) [34]. Increased proliferation occurs as a result of extracting low-molecular-weight, physiologically active peptides from soybean protein fermented with *L. rhamnosus* and processing them in fibroblasts at different concentrations [35]. Therefore, the main functional ingredient derived from soybeans exhibits low toxicity and increases the proliferation of human dermal fibroblast cells.

#### 3.2.2. Cytotoxicities of Soymilk Fractions in HaCaT Keratinocytes

**Figure 3:**
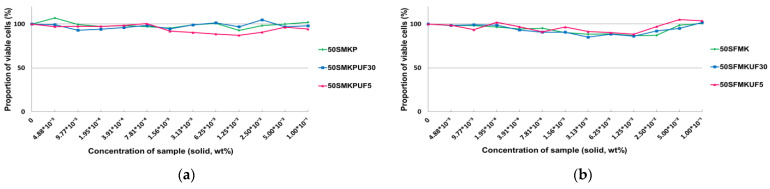
Cytotoxicities of various soymilk fractions in HaCaT keratinocytes. (**a**) 50SMKP, 50SMKPUF30, and 50SMKPUF5. (**b**) 50SFMK, 50SFMKUF30, and 50SFMKUF5.

To determine their cytotoxicities, immortalized HaCaT keratinocytes were treated with various concentrations of the soymilk fractions (Figure S3). Even at higher concentrations of the sample, the viability of cells remained high at 0.1% concentration, with 50SFMKUF5 at 103.71%, 50SMKP at 102.02%, 50SFMKUF30 at 101.83%, 50SPMK at 101.45%, 100SMK at 101.41%, 50SFMK at 100.7%, and 100SPMK at 100.23% (Figure 3). Additionally, 100SMKFUF5, which showed the highest toxicity, had a cell viability of 59.63%, indicating that approximately 40% of the cell population was killed compared to that in the control. Kim et al. [35] reported no cytotoxicity in HaCaT keratinocytes treated with various concentrations of soybean peptides fermented with *L. rhamnosus*, even at a high concentration of 0.2%.

Overall, proliferation increased when samples were treated with fibroblast cells, but toxicity was higher when HaCaT cells were treated with the 100% soymilk fraction than when treated with 50% soymilk. Based on these results, the anti-aging effects of the fractions prepared with 50% soymilk were determined by measuring the amount of type 1 procollagen and TNF-α produced.

### 3.3. Production of Type 1 Procollagen in Fibroblasts

**Figure 4:**
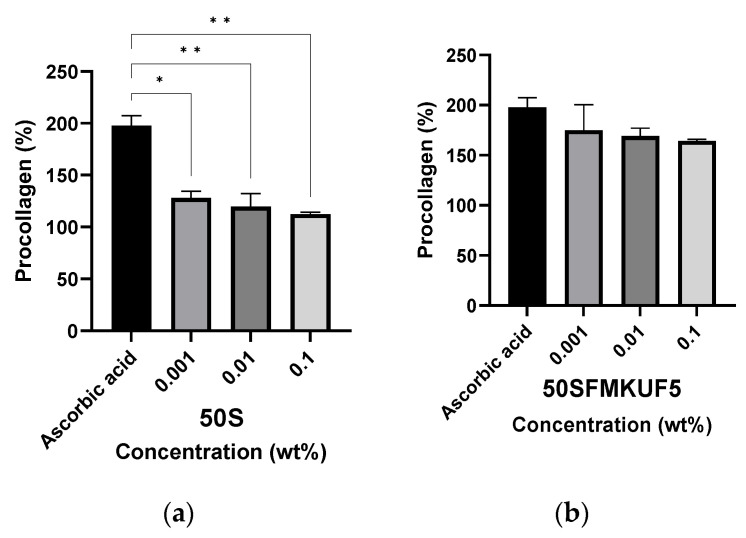
Production of type 1 procollagen in ultraviolet B (UVB)-irradiated human dermal fibroblasts treated with (**a**) 50S and (**b**) 50SFMKUF5. The mean ± standard deviation (SD) values of three independent experiments were analyzed using a two-way analysis of variance (ANOVA) and Dunnett’s test (* *p* < 0.05 and ** *p* < 0.01).

UV exposure causes collagen degradation in the dermis, which is one of the main causes of wrinkles [36]. To measure the inhibitory effect of the sample on photoaging, human dermal fibroblasts were exposed to UVB, and the amount of newly generated procollagen produced during cell growth was measured. After UVB irradiation of the fibroblasts, the untreated control was set to 100%, ascorbic acid was used as the positive control, and the soymilk fractions were used to treat cells at concentrations of 0.1%, 0.01%, and 0.001% (Figure 4). When fibroblasts were treated with ascorbic acid, the production of procollagen was 197.76%. Procollagen production in cells treated with 50% soymilk was significantly lower than that in cells treated with ascorbic acid (Figure 4a). When 50SFMKUF5, the soymilk fraction obtained by fermenting 50% flavourzyme-treated soymilk with lactic acid bacteria and fractionating by 5 kDa UF, was treated with fibroblast cells, the production of procollagen was confirmed to be 164.23%. No significant differences were observed in the results of the treatment with ascorbic acid and 50SFMKUF5 at different concentrations, as shown in Figure 4b. By contrast, when the fibroblasts were treated with 0.1% 50SPMKUF5, a fermented soymilk fraction treated with protamex, the production of procollagen was the lowest, at 94.97%, which was a two-fold difference from the positive control (Figure S4). When collagen hydrolysate was prepared by treatment with commercial proteases (flavourzyme and protamex), the protein recovery and free amino group production of the collagen hydrolysate treated with flavourzyme were higher than those treated with protamex [37]. Low-molecular-weight fraction of soybean concentrate treated with flavourzyme exerts antioxidant activity [38]; however, there have been no reports on the production of procollagen in the low-molecular-weight fraction obtained via fermentation with commercial proteases and lactic acid bacteria.

### 3.4. Inhibition of TNF-α Production in HaCaT Keratinocytes

**Figure 5:**
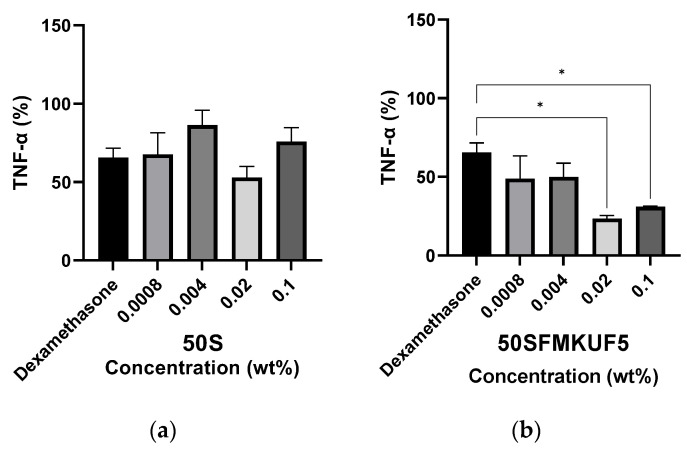
Inhibition of tumor necrosis factor (TNF)-α production in UVB-irradiated HaCaT keratinocytes treated with (**a**) 50S and (**b**) 50SFMKUF5. The mean ± SD values of three independent experiments were analyzed using two-way ANOVA and Dunnett’s test (* *p* < 0.05).

Exposure to UV radiation induces the release of pro-inflammatory mediators from skin cells, causing the infiltration and activation of immune cells [11]. The photoprotection of the skin is likely attributable to the antioxidant, anti-inflammatory, and anticarcinogenic properties of certain compounds [39]. In this study, the inflammation-alleviating effect of soymilk fractions on UVB-irradiated HaCaT keratinocytes was measured by assessing the amount of TNF-α, an indicator of inflammation, produced in response to treatment with soymilk fractions. After UVB irradiation of HaCaT keratinocytes, the untreated control was set to 100%, dexamethasone was used as the positive control, and the soymilk fractions were treated with 0.1, 0.02, 0.004, and 0.0008% cells (Figure 5 and Figure S5). The production of TNF-α in HaCaT keratinocytes treated with dexamethasone was measured at 65.58%. The amount of TNF-α produced in the cells treated with 50SMKFUF5, 50SMKPUF5, 50SFMKUF30, 50SFMKUF5, and 50SPMKUF5 was lower than that in the positive control. This indicated that these soymilk fractions were more effective than dexamethasone in inhibiting TNF-α production. When HaCaT keratinocytes were treated with 50SPMKUF5 and 50SFMKUF5 concentrations of 0.2%, 23.23% and 23.44% of TNF-α were measured, respectively, indicating that inflammation was effectively inhibited. Although 50SPMKUF5 inhibited the production of TNF-α the most, it was excluded from the final selection because of its cytotoxicity, as determined in the cytotoxicity evaluation. The production of TNF-α is decreased in UVB-irradiated HaCaT keratinocytes treated with persimmon oligomeric proanthocyanidins [40] or the *Rhododendron weyrichii* flower extract [41]. Here, 50SFMKUF5 exerted anti-inflammatory effects on UVB-irradiated cells by reducing the production of TNF-α.

In this study, 50SFMKUF5 was finally selected as the soymilk fraction with radical scavenging and anti-wrinkle effects based on efficacy evaluations, such as the DPPH radical scavenging activity, cell differentiation, MTT assays, and measurements of procollagen and TNF-α.

### 3.5. Characteristics of Functional Soymilk Fractions

#### 3.5.1. General and Reducing Sugars in Soymilk Fractions

Next, we confirmed the differences between the components of soymilk and the selected 50SFMKUF5. Table 3 shows the saccharide, carbohydrate, crude protein, crude lipid, and solid contents in soymilk. The crude protein, crude lipid, and solid contents of 50SFMKUF5 were 0.30 g, 0.00 g, and 1.37%, respectively. Compared to 100% soymilk, all general components showed lower values, possibly due to the effects of fermentation, enzyme treatment, and UF.

**Figure 6:**
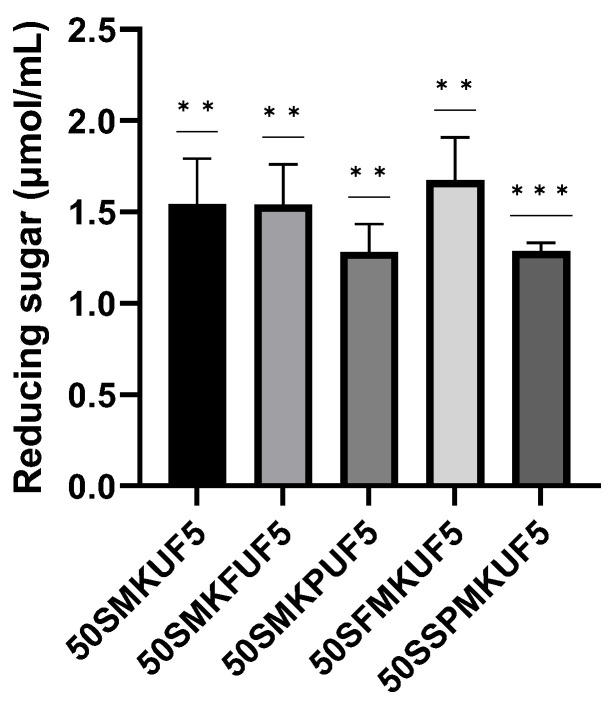
Reducing sugar contents of various soymilk fractions. The mean ± SD values of three independent experiments were analyzed using a *t*-test (** *p* < 0.01 and *** *p* < 0.001).

The results of reducing sugar analysis of the soymilk fractions are shown in Figure 6 There were no differences among the reducing sugars of the five fractions. However, 50SFMKUF5 exhibited a higher reducing sugar content of 1.67 µmol/mL than other soymilk fractions. The reducing sugar content of LAB-fermented soymilk decreases over time [42]. Additionally, in tofu residue treated with amylase, insoluble polysaccharides are converted to reducing sugars after enzyme treatment [43]. The results of this study showed that a significant portion of insoluble polysaccharides was converted by flavourzyme in 50SFMKUF5. The second highest amount of reducing equivalents was observed in 50SMKFUF5 cells treated with flavourzyme after fermentation.

#### 3.5.2. Free Amino Acid and Nitrogen Contents in Soymilk Fractions

During fermentation, the growth of lactic acid bacteria in soymilk relies on proteolysis to release amino acids [44]. In Table 4, the free amino acids of the fractions of less than 5 kDa obtained via UF were analyzed (50SMKUF5, 50SMKFUF5, 50SMKPUF5, 50SFMKUF5, and 50SPMKUF5). The total free amino acid (TA) content of 50SMKUF5 without enzyme treatment was the lowest, measuring 40.2 mg/100 g. On the other hand, the TA contents of 50SFMKUF5 and 50SPMKUF5, which were fermented after enzyme treatment, were higher than those of 50SMKFUF5 and 50SMKPUF5 treated with enzyme after fermentation. The TA content of 50SFMKUF5 was the highest at 67.3 mg/100 g, whereas that of 50SMKFUF5 was 46.8 mg/100 g. The free amino acid content of soymilk increases by 234.8% when fermented with five types of lactic acid bacteria and hydrolyzed with proteases [45]. Fermenting soymilk with *Lactobacillus delbrueckii* subsp. *Delbrueckii* increases the overall content of free amino acids [46]. These results showed that the TA content of the fraction treated with the enzyme later was higher than that of the fraction treated with the enzyme first because protease treatment of soymilk protein prior to fermentation allowed *L. paracasei* MK1 to cleave the protein more effectively.

Here, glutamic acid content accounted for 10.7% of the total free amino acids in 50SFMKUF5, which was lower compared to that reported in a previous study [47] on soymilk where the glutamic acid content was found to be 733 mg/100 mL, representing 19.1% of the total amino acids. Although the leucine and arginine contents in soymilk were 7.7 and 7.9%, respectively, in 50SFMKUF5, their amino acid contents were 16.6 and 17.7%, respectively. However, because the overall amino acid content was significantly reduced, many free amino acids were lost through various processes. The nutritionally important essential amino acid content was similar to that of TA. According to these results, when *L. paracasei* MK1 used soymilk protein in soymilk fermentation, enzymatic treatment resulted in better degradation of soymilk protein, suggesting that flavourzyme is more effective than the other enzymes.

Total nitrogen content provides an estimate of the number of peptides or proteins present in the sample, whereas the TCA-soluble nitrogen content estimates the nitrogen compound content when proteins undergo hydrolysis by proteases [48]. The total and TCA-soluble nitrogen contents of the soymilk fractions were measured to evaluate the changes in nitrogen content due to fermentation and enzyme treatment. The total nitrogen content of 50% soymilk was the highest at 2.82 mg N/100 mL, but its TCA-soluble nitrogen content was the lowest at 0.12 mg N/100 mL (Table 5). The total nitrogen content of the samples treated with the enzyme was higher than that of the untreated samples. Free amino acids, oligopeptides, amines, and other compounds comprising non-protein nitrogen (NPN) are an important group of cosmetic components owing to their technological and functional significance. These compounds play a significant role in cosmetic development, particularly in the formulation of skin care products. NPN possesses excellent moisturizing properties as well as an anti-aging effect. [49]. The content of NPN resulting from the enzyme treatment was evident through the TCA-soluble nitrogen content, and this result was also observed for 50SFMKUF5 and 50SPMKUF5, which were measured at 0.45 and 0.82 mg N/100 mL, respectively, showing a similar trend to the total nitrogen content.

### 3.6. Analysis of Peptides Derived from 50SFMKUF5

#### 3.6.1. Screening of Bioactive Compounds from 50SFMKUF5

**Figure 7:**
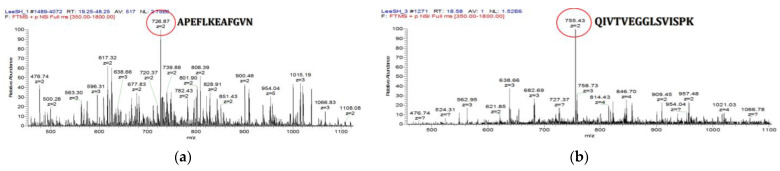
Liquid chromatography–tandem mass spectrometry (LC-MS/MS) chromatograms of 50SFMKUF5. (**a**) APEFLKEAFGVN and (**b**) QIVTVEGGLSVISPK.

Based on these results, 50SFMKUF5, a sample obtained by processing flavourzyme and fractionating soymilk fermented with *L. paracasei* MK1 into UF 5 kDa, was selected as the final fraction. Peptide mapping was performed using LC-MS/MS to search for biologically active compounds present in 50SFMKUF5. The results of the MS analysis are shown in Figure 7, and two major peaks, 726.87 and 755.43 m/z, were identified. The respective molecular weights were confirmed to be 1453.74 and 1510.86 Da, and the amino acid sequences were shown as APEFLKEAFGVN (APE) and QIVTVEGGLSVISPK (QIV), respectively. The APE and QIV were found to be glycinin G2 (NCBI NP_001235810.1) and glycinin G4 (NCBI NP_001238008.1), respectively, as a result of searching using NCBI BLAST, and the full amino acid sequences of glycinin G2 and G4 are shown in Figures S6 and S7, respectively. Soymilk fermented with *L. plantarum* C2 was fractionated at 10, 5, and 3 kDa to confirm the ACE-inhibitory effect, and as a result of LC/MS-MS, a GFAPEFLKEAFGVN sequence of 736.3768 m/z at a retention time of 65.99 min was found [50]. As a result of examining the peptide components present in milk with pea protein added, there have been reports that the measured contents of LQESVIVEISK, FYLAGNQEQEFLK, SQSDNFEYVSFK, and QIVTVEGGLSVISPK were high [51]. Previous studies have reported that conglycinin and glycinin, the two main protein compounds in soybeans, can yield soy-derived peptides with antioxidant activity that are more effective than those derived from conglycinin [52]. To verify the functionality of the glycinin G2- and G4-derived peptides identified in this study, APE and QIV were synthesized and tested on human dermal fibroblasts, HaCaT keratinocytes, and melanoma cells to assess their ability to stimulate procollagen production, inhibit TNF-α, and reduce melanin production.

#### 3.6.2. Production of Type 1 Procollagen in Fibroblasts after Peptide Treatment

**Figure 8:**
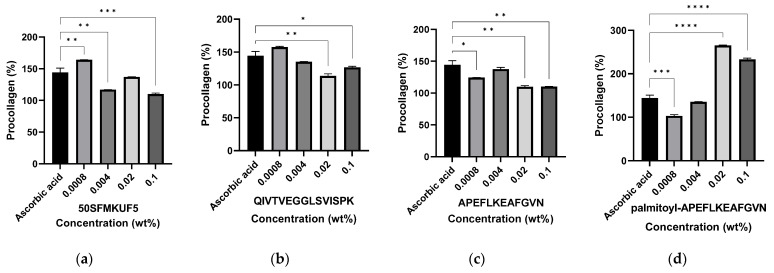
Production of type 1 procollagen based on the peptide concentration in UVB-irradiated fibroblast cells. Cells were treated with (**a**) 50SFMKUF5, (**b**) QIVTVEGGLSVISPK, (**c**) APEFLKEAFGVN, (**d**) palmitoyl-APEFLKEAFGVN. The mean ± SD values of three independent experiments were analyzed using two-way ANOVA and Dunnett’s test (* *p* < 0.05, ** *p* < 0.01, *** *p* < 0.001, and **** *p* < 0.0001).

As the molecular weight of peptides is too high (500 Da) to cross the skin barrier, they are not suitable for absorption through or into the skin [53]. Here, the molecular weights of APE and QIV were 1453.74 and 1510.86 Da, respectively, which is much higher than 500 Da. To confirm the anti-wrinkle-like effect of peptides, they must pass through the stratum corneum, which is the skin barrier, and the epidermis to reach the dermis at an optimal concentration [54]. To improve the permeability of peptides, the combination of fatty acids, such as palmitic acids, and peptides holds significant potential as an effective approach for enhancing the permeability and stability of peptides [55]. In this study, UVB-irradiated fibroblasts were treated with 50SFMKUF5, a soymilk fraction, APE, QIV, and Pal-APE. Pal-APE was synthesized by attaching palmitic acid to APE. The objective of this study was to determine the amount of type 1 procollagen production (Figure 8). Cells not treated with anything after UVB irradiation was used as a control, and 50 µM of ascorbic acid was used as a positive control. As the concentrations of 50SFMKUF5, APE, and QIV increased in fibroblasts, the amount of procollagen produced significantly decreased compared to the positive control. Contrary to previous results, when cells were treated with Pal-APE at concentrations of 0.02 and 0.1%, the amount of procollagen produced was significantly increased compared to the positive control. When passing through the skin, palmitoyl-binding polypeptides have been reported to have 100–1000 times improved permeability compared to general polypeptides [56]. In addition, when Lys-Thr-Thr-Lys-Ser (KTTKS), derived from a fragment of type 1 procollagen, was modified with palmitoyl pentapeptide-4 (Pal-KTTKS-OH), its stability and permeability were improved and became better than those of KTTKS [57]. Pal-KTTKS was one of the first polypeptides used in cosmetics, and its application to the skin of 49 women for 4 months significantly improved their skin roughness, wrinkle volume, and wrinkle depth [58]. These results confirmed that the skin permeability of Pal-APE was better than that of 50SFMKUF5, a soymilk-derived fraction, and that of synthesized APE and QIV. This improvement led to enhanced procollagen production.

#### 3.6.3. Inhibition of TNF-α Production in HaCaT Keratinocytes after Peptide Treatment

**Figure 9:**
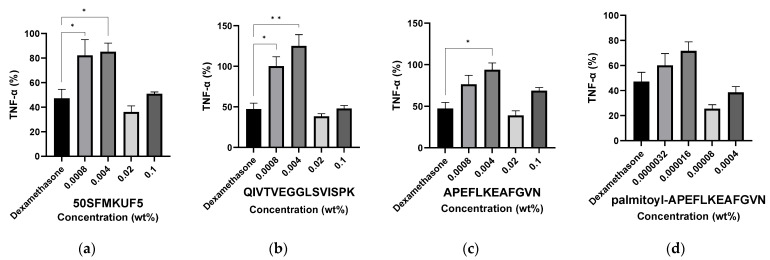
TNF-α inhibition according to the peptide concentration in UVB-irradiated HaCaT keratinocytes. Cells were treated with (**a**) 50SFMKUF5, (**b**) QIVTVEGGLSVISPK, (**c**) APEFLKEAFGVN, and (**d**) palmitoyl-APEFLKEAFGVN. The mean ± SD values of three independent experiments were analyzed using two-way ANOVA and Dunnett’s test (* *p* < 0.05 and ** *p* < 0.01).

To assess the degree of TNF-a production inhibition, HaCaT keratinocytes were treated with 50SFMKUF5 and three synthetic peptides at varying concentrations. As a positive control, the anti-inflammatory drug dexamethasone (1 µM) was used for treatment and the inhibition rate of TNF-α was compared (Figure 9). Notably, the production of TNF-α was significantly higher in cells treated with low concentrations (0.0008 and 0.004%) of 50SFMKUF5, QIV, APE, and Pal-APE than in the positive control. When the cells were treated with 0.02 and 0.1% 50SFMKUF5, QIV, and APE, the production of TNF-α was not significantly different from that in the positive control. Furthermore, no significant difference in the amount of TNF-a produced from the positive control was observed at all concentrations when cells were treated with Pal-APE. These results indicated that 50SFMKUF5, QIV, APE, and Pal-APE exhibit anti-inflammatory effects. Specifically, Pal-APE exerted anti-inflammatory effects at all tested concentrations. Among the soy proteins, peptide FLV, derived from β-conglycinin, inhibits the synthesis of pro-inflammatory cytokines, such as TNF-α and IL-6, in 3T3-L1 adipocytes [59]. Additionally, peptide VPY, derived from glycinin, downregulates the level of pro-inflammatory cytokines, such as TNF-α, IL-6, and IL-1β, in Caco-2 intestinal epithelial cells and THP-1 macrophages [60]. Lunasin, a soy-derived peptide consisting of 43–44 amino acids, exerts anti-inflammatory and anticancer effects in RAW264.7 macrophages [61]. However, limited information is available on the anti-inflammatory effects of peptides derived from fermented soymilk fractions and palmitoylated peptides.

#### 3.6.4. Inhibition of Melanin Synthesis in B16F1 Melanoma Cells after Peptide Treatment

**Figure 10:**
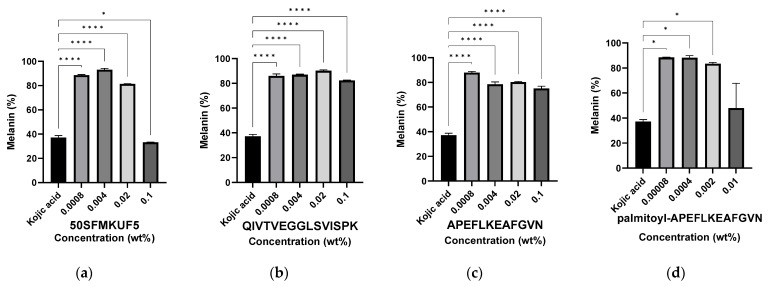
Melanin inhibition according to the peptide concentration in α-melanocyte-stimulating hormone (α-MSH)-treated B16F1 melanoma cells. Cells were treated with (**a**) 50SFMKUF5, (**b**) QIVTVEGGLSVISPK, (**c**) APEFLKEAFGVN, and (**d**) palmitoyl-APEFLKEAFGVN. The mean ± SD values of three independent experiments were analyzed using two-way ANOVA and Dunnett’s test (* *p* < 0.05 and **** *p* < 0.0001).

Hyperpigmentation of the skin occurs when an excessive amount of melanin accumulates in melanocytes within skin cells, primarily due to environmental factors, such as α-melanocyte-stimulating hormone (α-MSH) treatment, UV radiation, cAMP-elevating agents, estrogen, hormonal factors, and genetic factors. Melanin is subsequently transported to the keratinocytes and accumulates in the epithelial layer of the skin [62,63]. Melanin promotes cell death due to the toxicity caused by pigmentation and melanin precursors in terms of beauty. Various substances, such as hydroquinone, arbutin, kojic acid, and vitamin C, prevent melanin deposition, but their actual clinical effects remain unclear [64,65,66]. In this study, melanin production was induced by treating B16F1 melanoma cells with 5 nM of α-MSH, and 1.6 mM kojic acid was used as a positive control. Treatment with kojic acid, the positive control, resulted in a 37.25% reduction in melanin production (Figure 10). Neither QIV nor APE exhibited any inhibitory effects on melanogenesis at any concentration. However, when 50SFMKUF5 was treated with 0.1%, it was measured as 33.26%, which was lower than the positive control, and melanin formation was significantly reduced, but when Pal-APE was treated with 0.01%, melanin formation was reduced by 47.97%; however, the difference was not significant. In a parallel treatment study involving a mixture of soy- and collagen-derived peptides and *Chrysanthemum morifolium* water-soluble extract, a significant reduction of 46.2% in melanogenesis was observed [67]. Additionally, a separate report revealed that treatment with soybean cell culture extract inhibits melanin synthesis in α-MSH-induced B16F10 melanoma cells in a dose-dependent manner [68]. In this study, 50SFMKUF5 and Pal-APE soy-derived peptides demonstrated the potential to inhibit melanin synthesis, which is consistent with the findings of previous studies.

## 4. Conclusions

In this study, we evaluated the radical scavenging activity and anti-wrinkle effects of the 30 and 5 kDa fractions of soymilk fermented with *L. paracasei* MK1 and subsequently treated with commercial proteases (flavourzyme or protamex). Among the various fractions tested, the fraction obtained by fermenting flavourzyme-treated soymilk with *L. paracasei* MK1 followed by ultrafiltration at 5 kDa (50SFMKUF5) exhibited the best characteristics. This fraction exhibited high radical scavenging capacity, promoted the proliferation and type 1 procollagen production in fibroblasts, and reduced the cytotoxicity and TNF-α production in HaCaT keratinocytes. Moreover, APEFLKEAFGVN, QIVTVEGGLSVISPK, and Pal-APE peptides derived from 50SFMKUF5 exerted anti-inflammatory effects by reducing the TNF-α levels in cells. Both 50SFMKUF5 and Pal-APE inhibited melanin production in melanoma cells, and Pal-APE specifically increased the type 1 procollagen production in cells. Despite its high molecular weight, Pal-APE derived from 50SFMKUF5 exhibited potent anti-inflammatory effects, inhibited melanin production, and induced type 1 procollagen production. Our findings highlight the potential benefits of Pal-APE to human skin.

## Figures and Tables

**Table 1:** Preparation methods of various samples used in this study.

Sample Name	Flow Chart of Fermentation or/and Fractionation
50S	50% Soymilk
50SMK	50% Soymilk→Fermented with *L. paracasei* MK1
50SMKUF30	50% Soymilk→Fermented with *L. paracasei* MK1→UF 30 kDa
50SMKUF5	50% Soymilk→Fermented with *L. paracasei* MK1→UF 30 kDa→UF 5 kDa
SF	100% Soymilk→Flavourzyme
SP	100% Soymilk→Protamex
50SMKF	50% Soymilk→Fermented with *L. paracasei* MK1→Flavourzyme
50SMKFUF30	50% Soymilk→Fermented with *L. paracasei* MK1→Flavourzyme→UF 30 kDa
50SMKFUF5	50% Soymilk→Fermented with *L. paracasei* MK1→Flavourzyme→UF 30 kDa→UF 5 kDa
50SMKP	50% Soymilk→Fermented with *L. paracasei* MK1→Protamex
50SMKPUF30	50% Soymilk→Fermented with *L. paracasei* MK1→Protamex→UF 30 kDa
50SMKPUF5	50% Soymilk→Fermented with *L. paracasei* MK1→Protamex→UF 30 kDa→UF 5 kDa
50SFMK	50% Soymilk→Flavourzyme→Fermented with *L. paracasei* MK1
50SFMKUF30	50% Soymilk→Flavourzyme→Fermented with *L. paracasei* MK1→UF 30 kDa
50SFMKUF5	50% Soymilk→Flavourzyme→Fermented with *L. paracasei* MK1→UF 30 kDa→UF 5 kDa
50SPMK	50% Soymilk→Protamex→Fermented with *L. paracasei* MK1
50SPMKUF30	50% Soymilk→Protamex→Fermented with *L. paracasei* MK1→UF 30 kDa
50SPMKUF5	50% Soymilk→Protamex→Fermented with *L. paracasei* MK1→UF 30 kDa→UF 5 kDa
100S	100% Soymilk
100SMK	100% Soymilk→Fermented with *L. paracasei* MK1
100SMKUF30	100% Soymilk→Fermented with *L. paracasei* MK1→UF 30 kDa
100SMKUF5	100% Soymilk→Fermented with *L. paracasei* MK1→UF 30 kDa→UF 5 kDa
100SMKF	100% Soymilk→Fermented with *L. paracasei* MK1→Flavourzyme
100SMKFUF30	100% Soymilk→Fermented with *L. paracasei* MK1→Flavourzyme→UF 30 kDa
100SMKFUF5	100% Soymilk→Fermented with *L. paracasei* MK1→Flavourzyme→UF 30 kDa→UF5 kDa
100SMKP	100% Soymilk→Fermented with *L. paracasei* MK1→Protamex
100SMKPUF30	100% Soymilk→Fermented with *L. paracasei* MK1→Protamex→UF 30 kDa
100SMKPUF5	100% Soymilk→Fermented with *L. paracasei* MK1→Protamex→UF 30 kDa→UF 5 kDa
100SFMK	100% Soymilk→Flavourzyme→Fermented with *L. paracasei* MK1
100SFMKUF30	100% Soymilk→Flavourzyme→Fermented with *L. paracasei* MK1→UF 30 kDa
100SFMKUF5	100% Soymilk→Flavourzyme→Fermented with *L. paracasei* MK1→UF 30 kDa→UF 5 kDa
100SPMK	100% Soymilk→Protamex→Fermented with *L. paracasei* MK1
100SPMKUF30	100% Soymilk→Protamex→Fermented with *L. paracasei* MK1→UF 30 kDa
100SPMKUF5	100% Soymilk→Protamex→Fermented with *L. paracasei* MK1→UF 30 kDa→UF 5 kDa

**Table 2:** Half-maximal inhibitory concentration (IC_50_) values of soymilk fractions.

Sample	IC_50_ (Solid, wt%)	Sample	IC_50_ (Solid, wt%)
50S	1.24	100S	ND ^1^
50SMK	0.67	100SMK	0.86
50SMKUF30	0.81	100SMKUF30	0.81
50SMKUF5	0.75	100SMKUF5	0.91
SF	ND1	100SMKF	0.91
SP	ND	100SMKFUF30	0.86
50SMKF	0.86	100SMKFUF5	0.95
50SMKFUF30	0.78	100SMKP	0.86
50SMKFUF5	0.86	100SMKPUF30	0.87
50SMKP	0.78	100SMKPUF5	0.91
50SMKPUF30	0.86	100SFMK	0.75
50SMKPUF5	0.91	100SFMKUF30	0.99
50SFMK	0.81	100SFMKUF5	0.86
50SFMKUF30	0.78	100SPMK	0.78
50SFMKUF5	0.86	100SPMKUF30	0.91
50SPMK	0.78	100SPMKUF5	0.91
50SPMKUF30	0.99		
50SPMKUF5	0.87		

^1^ ND, not detected.

**Table 3:** General components of 100% soymilk and 50SMKUF5.

Component	Content	
	100% Soymilk	50SMKUF5
Saccharide (g/100 g)	0.71	ND ^1^
Carbohydrate (g/100g)	3.40	ND
Crude protein (g/100 g)	4.30	0.30
Crude lipid (g/100 g)	2.40	0.00
Solid (%)	10.80	1.37

^1^ ND, not detected.

**Table 4:** Compositions of free amino acids in various soymilk fractions.

Amino Acid	Concentration of Free Amino Acid (mg/100 g, %)				
	50SMKUF5	50SMKFUF5	50SMKPUF5	50SFMKUF5	50SPMKUF5
Aspartic acid	1.8 (4.5)	1.9 (4.1)	1.9 (4.5)	2.6 (3.9)	1.2 (2.8)
Glutamic acid	12.5 (31.0)	13.3 (28.4)	11.2 (27.0)	7.2 (10.7)	4.6 (10.5)
Asparagine	0.4 (1.0)	0.6 (1.2)	0.4 (0.9)	1.0 (1.5)	0.2 (0.4)
Serine	0.2 (0.6)	0.5 (1.1)	0.3 (0.6)	0.2 (0.3)	0.1 (0.2)
Glutamine	0.0 (0.0)	0.5 (1.1)	0.0 (0.0)	0.0 (0.0)	0.0 (0.0)
Glycine	0.8 (2.0)	0.7 (1.5)	0.7 (1.7)	0.6 (0.8)	1.2 (2.7)
Histidine	1.6 (3.9)	1.6 (3.4)	1.7 (4.2)	1.3 (2.0)	1.2 (2.8)
Arginine	10.9 (27.0)	10.2 (21.8)	10.7 (25.8)	11.9 (17.7)	9.6 (22.1)
Threonine	0.3 (0.6)	0.7 (1.6)	0.4 (1.0)	0.5 (0.8)	0.5 (1.1)
Alanine	2.4 (6.0)	2.5 (5.3)	2.5 (6.1)	2.9 (4.3)	3.1 (7.1)
Proline	2.9 (7.2)	2.7 (5.9)	3.8 (9.1)	1.5 (2.2)	1.0 (2.3)
Tyrosine	1.5 (3.7)	0.7 (1.6)	1.5 (3.6)	3.1 (4.5)	1.4 (3.2)
Valine	0.3 (0.7)	0.7 (1.4)	0.3 (0.7)	2.2 (3.3)	1.7 (4.0)
Methionine	0.0 (0.0)	0.7 (1.6)	0.0 (0.0)	0.3 (0.5)	0.5 (1.2)
Cysteine	0.0 (0.0)	0.1 (0.3)	0.1 (0.2)	0.1 (0.2)	0.0 (0.0)
Isoleucine	0.0 (0.0)	0.6 (1.2)	0.2 (0.4)	2.4 (3.6)	2.1 (4.8)
Leucine	0.4 (0.9)	2.1 (4.5)	0.6 (1.4)	11.2 (16.6)	4.7 (10.8)
Phenylalanine	0.6 (1.5)	2.4 (5.1)	1.0 (2.4)	15.0 (22.3)	6.4 (14.6)
Tryptophan	2.7 (6.7)	2.6 (5.6)	3.0 (7.3)	2.7 (4.0)	3.9 (8.9)
Lysine	1.0 (2.5)	1.7 (3.6)	1.2 (3.0)	0.6 (0.8)	0.2 (0.4)
TA ^1^	40.2 (100)	46.8 (100)	41.4 (100)	67.3 (100)	43.6 (100)
EA ^2^	6.9 (16.8)	13.1 (28.0)	8.4 (20.4)	36.2 (53.9)	21.2 (43.6)

^1^ TA, total free amino acid. ^2^ EA, essential amino acid (Thr + Val + Met + Ile + Leu + Phe + Lys + Trp + His).

**Table 5:** Changes in the trichloroacetic acid (TCA)-soluble nitrogen concentration in various soymilk fractions.

Soymilk Fraction	Total Nitrogen (mg N/100 mL)	TCA-Soluble Nitrogen (mg N/100 mL)	TN/SN (%)
50S	2.82 ± 0.17	0.12 ± 0.03	4.39
50SMKUF5	0.34 ± 0.07	0.12 ± 0.05	36.17
50SMKFUF5	0.60 ± 0.14	0.36 ± 0.03	59.59
50SMKPUF5	0.40 ± 0.09	0.23 ± 0.01	58.02
50SFMKUF5	1.22 ± 0.23	0.45 ± 0.02	36.93
50SPMKUF5	1.60 ± 0.22	0.82 ± 0.06	51.16

Data are presented as mean ± SD of three independent experiments.

## Data Availability

All data generated in this study are presented in this article and the Supplementary Materials file.

## Supplementary Materials

The following supporting information can be downloaded at: https://www.mdpi.com/article/10.3390/antiox12071392/s1, Table S1: Instrument and operating condition of HPLC for the analysis of free amino acids; Table S2: Instrument and operating condition of LC/MS-MS for the analysis of peptides; Figure S1: DPPH radical scavenging activity of soymilk fractions; Figure S2: Effect of soymilk fractions on the viability of human dermal fibroblasts; Figure S3: Effect of soymilk fractions on the cytotoxicity of HaCaT keratinocytes; Figure S4: Production of type 1 procollagen in UVB-irradiated human dermal fibroblasts treated with soymilk fractions; Figure S5: Inhibition of TNF-α in UVB-irradiated HaCaT keratinocytes treated with soymilk fractions; Figure S6: Amino acid sequence of glycinin G2; Figure S7: Amino acid sequence of glycinin G4; Figure S8: Effect of peptides on the viability of human dermal fibroblasts; Figure S9: Cytotoxic effects of the peptides on HaCaT keratinocytes; Figure S10: Cytotoxic effects of the peptides on B16F1 melanoma cells.
