# Supplementary material for: The Radical Scavenging Activities and Anti-Wrinkle Effects of Soymilk Fractions Fermented with Lacticaseibacillus paracasei MK1 and Their Derived Peptides
Source: Antioxidants (Basel). 2023 Jul 6;12(7):1392. doi: 10.3390/antiox12071392 (PMC10376689; doi:10.3390/antiox12071392)
Supplement: Supplementary file 1 [file antioxidants-12-01392-s001.zip › antioxidants-2460665-supplementary.pdf]

Table S1. Instrument and operating condition of HPLC for the analysis of free amino acids

	Condition			
System	Waters 510 HPLC pump			
	Waters Gradient controller			
	Waters 717 automatic sampler			
Column	Waters pico-tag column (3.9×300 mm, 4 μm)			
Detector	Waters 2487 UV detector, 254 nm			
Data analysis	Empower 2 software			
Gradient condition	Time(min)	Flow	%A*	%B
	Initial	1.0	100	0
	9.0	1.0	86	14
	9.2	1.0	80	20
	17.5	1.0	54	46
	17.7	1.0	0	100
	21.0	1.0	100	0
	24.0	1.0	100	0
	25.0	1.0	100	0
	*%A: 140 mM sodium acetate (6% acetonitrile)			
	%B: 60% acetonitrile			

Table S2. Instrument and operating condition of LC/MS-MS for the analysis of peptide

<table>
<tr><th></th><th>Condition</th></tr>
<tr><td>System</td><td>Auto switching nano pump and autosampler (Tempo nano LC system, MDC SCIEX, Canada)</td></tr>
<tr><td>Column</td><td>Zorbax 300SB-C18 trap column (300 μm i.d×5 mm, 5 μm, 100 Å, Agilent Technologies, part number 5065-9913)
Zorbax 300SB-C18 capilary column (75 μm i.d × 150 mm, 3.5 μm, 100 Å, part number 5065-9911)
Hybrid Quadrupole-TOF MS/MS spectrometer (QStar Elite, Applied Biosystems, USA)</td></tr>
<tr><td>Detector</td><td>Analyst QS 2.0 software (Applied Biosystems, USA)</td></tr>
<tr><td>Data analysis

Gradient condition</td><td>
<table>
<tr><th>Time (min)</th><th>Flow (nL/min)</th><th>%A*</th><th>%B</th></tr>
<tr><td>Initial</td><td>5,000</td><td>98</td><td>2</td></tr>
<tr><td>6</td><td>300</td><td>65</td><td>35</td></tr>
<tr><td>36</td><td>300</td><td>10</td><td>90</td></tr>
<tr><td>46</td><td>300</td><td>10</td><td>90</td></tr>
<tr><td>51</td><td>300</td><td>95</td><td>5</td></tr>
<tr><td>66</td><td>300</td><td>95</td><td>5</td></tr>
</table>
*%A: Water/acetonitrile (98:2, v/v), 0.1% formic acid
%B: Water/acetonitrile (2:98, v/v), 0.1% formic acid</td></tr>
</table>

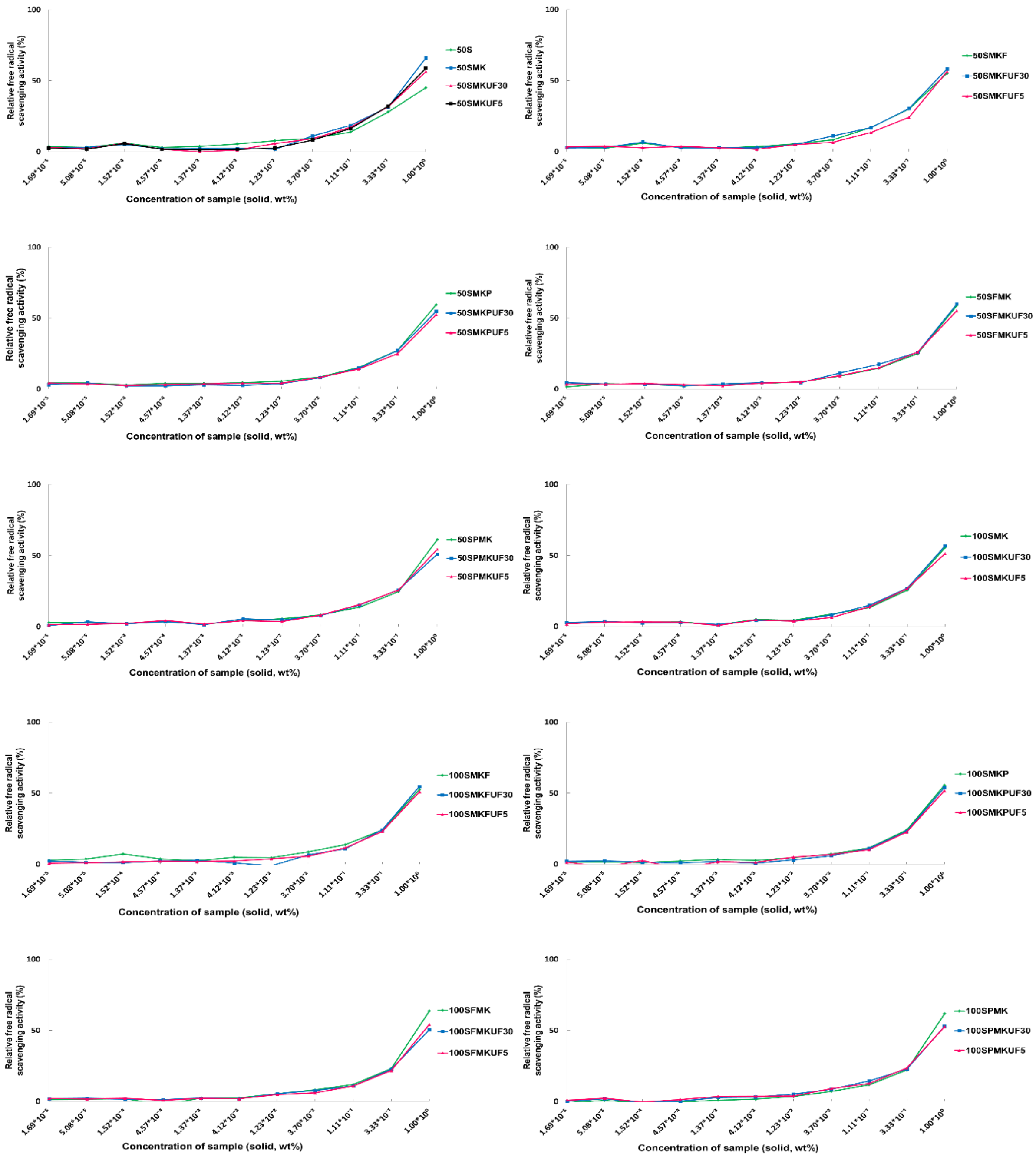

Figure S1. DPPH radical scavenging activity of soymilk fractions.

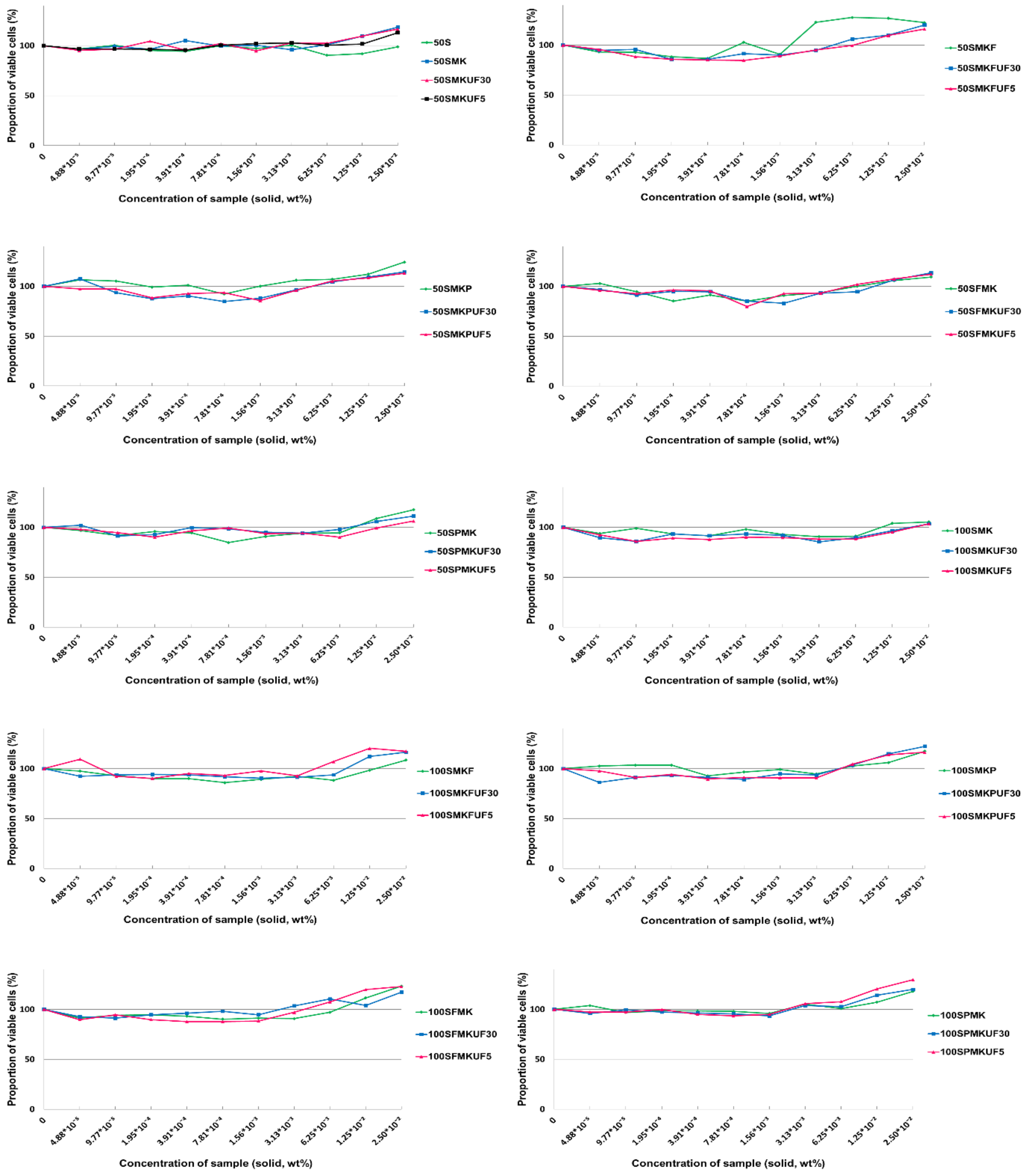

Figure S2. Effect of soymilk fractions on the viability of human dermal fibroblasts.

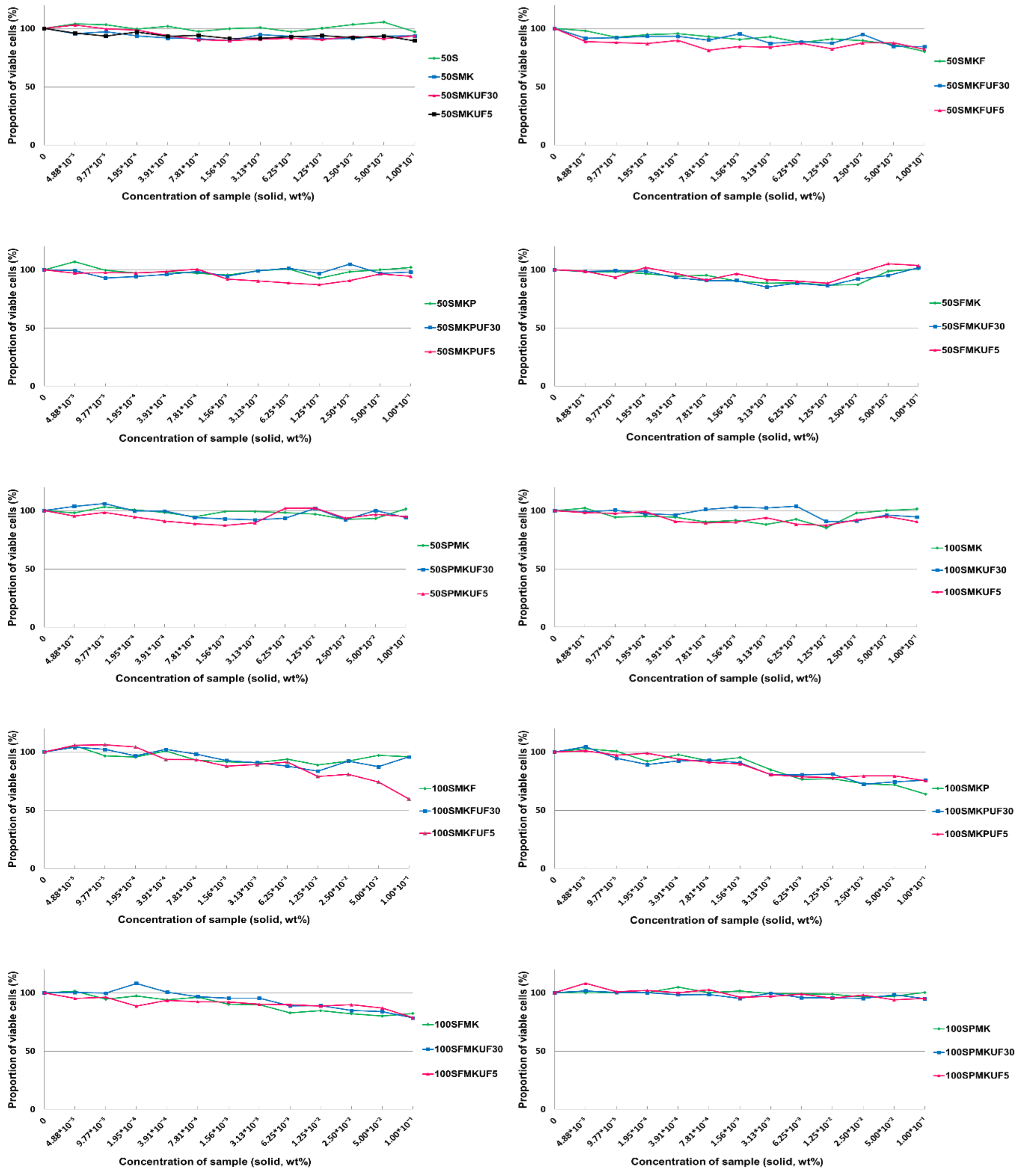

Figure S3. Effect of soymilk fractions on the cytotoxicity of HaCaT keratinocytes.

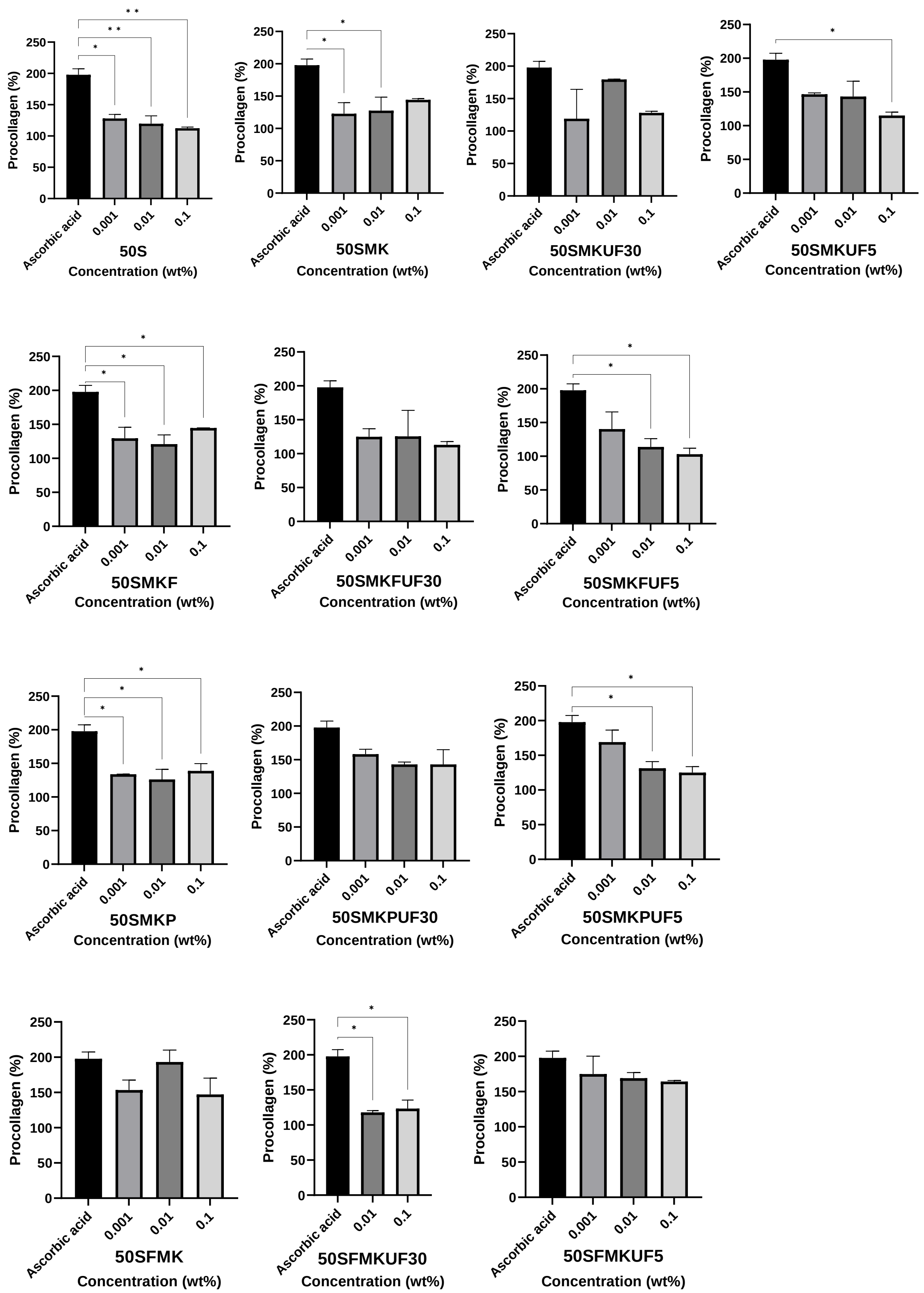

Procollagen (%)
250
200
150
100
50
0
Ascorbic acid
0.001
0.01
0.1
*
* *
50S
Concentration (wt%)
50SMK
50SMKUF30
50SMKUF5
50SMKF
50SMKFUF30
50SMKFUF5
50SMKP
50SMKPUF30
50SMKPUF5
50SFMK
50SFMKUF30
50SFMKUF5

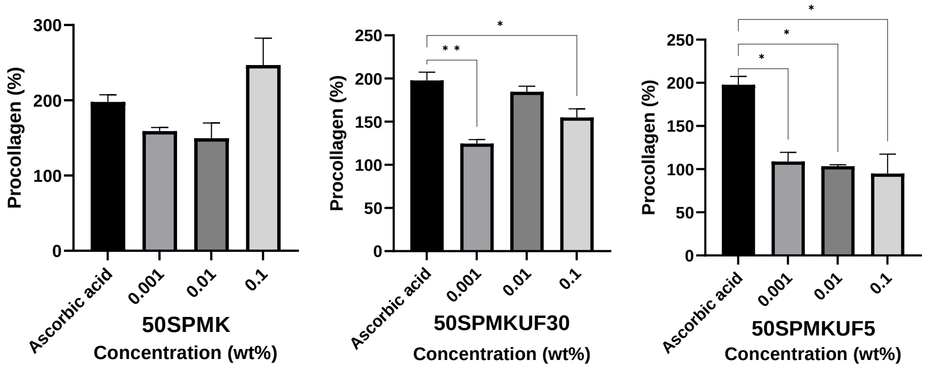

Figure S4. Production of type-1-procollagen in UVB-irradiated human dermal fibroblasts treated with soymilk fractions. The mean ± SD values of the results were analyzed using two-way ANOVA and Dunnet's test ($^{*}p$<0.05, $^{**}p$<0.01).

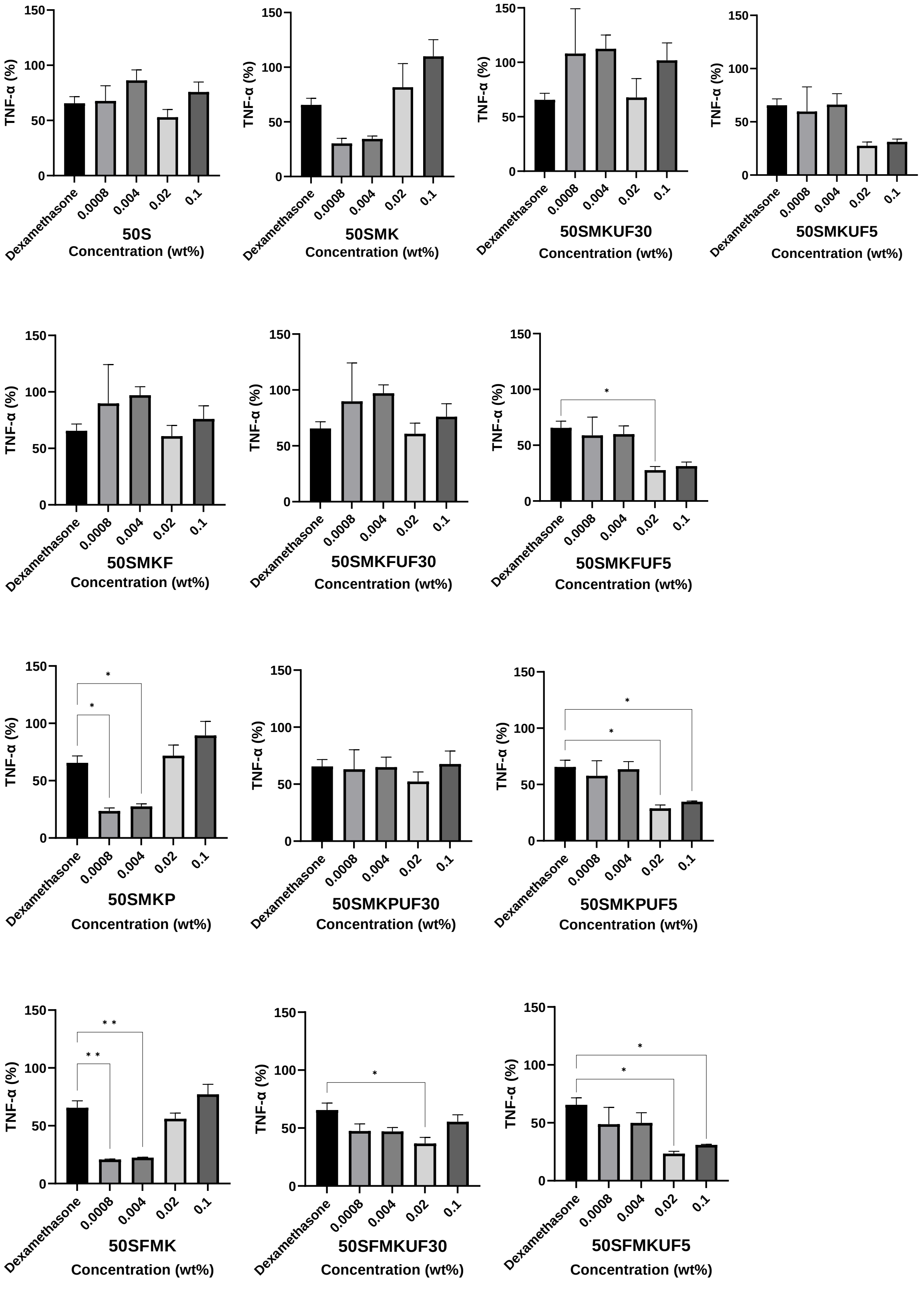

TNF-α (%)
150
100
50
0
Dexamethasone
0.0008
0.004
0.02
0.1
50S
Concentration (wt%)
50SMK
Concentration (wt%)
50SMKUF30
Concentration (wt%)
50SMKUF5
Concentration (wt%)
50SMKF
Concentration (wt%)
50SMKFUF30
Concentration (wt%)
50SMKFUF5
Concentration (wt%)
50SMKP
Concentration (wt%)
50SMKPUF30
Concentration (wt%)
50SMKPUF5
Concentration (wt%)
50SFMK
Concentration (wt%)
50SFMKUF30
Concentration (wt%)
50SFMKUF5
Concentration (wt%)

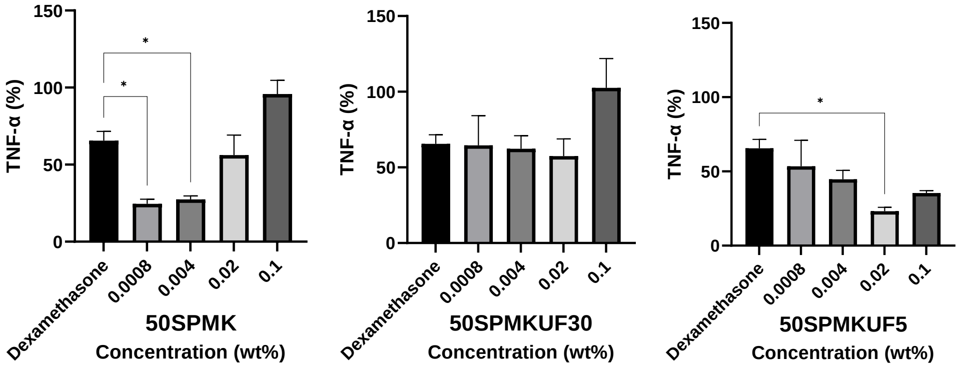

Figure S5. Inhibition of TNF-*α* in UVB-irradiated HaCaT keratinocytes treated with soymilk fractions. The mean ± SD values of the results were analyzed using two-way ANOVA and Dunnet's test ($^{*}p$<0.05, $^{**}p$<0.01).

MAKLVLSLCF LLFSGCFALR EQAQQNECQI QKLNALKPDN RIESEGGFIE TWNPNNKPFQ CAGVALSRCT LNRNALRRPS YTNGPQEIYI QQGNGIFGMI FPGCPSTYQE PQESQQRGRS QRPQDRHQKV HRFREGDLIA VPTGVAWWMY NNEDTPVVAV SIIDTNSLEN QLDQMPRRFY LAGNQEQEFL KYQQQQQGGS QSQKGKQQEE ENEGSNILSG FAPEFLKEAF GVNMQIVRNL QGENEEEDSG AIVTVKGGLR VTAPAMRKPQ QEEDDDDEEE QPQCVETDKG CQRQSKRSRN GIDETICTMR LRQNIGQNSS PDIYNPQAGS ITTATSLDFP ALWLLKLSAQ YGSLRKNAMF VPHYTLNANS IIYALNGRAL VQVVNCNGER VFDGELQEGG VLIVPQNFAV AAKSQSDNFE YVSFKTNDRP SIGNLAGANS LLNALPEEVI QHTFNLKSQQ ARQVKNNNPF SFLVPPQESQ RRAVA

Figure S6. Amino acid sequence of glycinin G2 (NCBI NP_001235810.1).

MGKPFTLSLS SLCLLLLSSA CFAISSSKLN ECQLNNLNAL EPDHRVESEG GLIQTWNSQH PELKCAGVTV SKLTLNRNGL HLPSYSPYPR MIIIAQGKGA LGVAIPGCPE TFEEPQEQSN RRGSRSQKQQ LQDSHQKIRH FNEGDVLVIP PGVPYWTYNT GDEPVVAISL LDTSNFNNQL DQTPRVFYLA GNPDIEYPET MQQQQQQKSH GGRKQGQHQQ EEEEEGGSVL SGFSKHFLAQ SFNTNEDIAE KLQSPDDERK QIVTVEGGLS VISPKWQEQQ DEDEDEDEDD EDEQIPSHPP RRPSHGKREQ DEDEDEDEDK PRPSRPSQGK REQDQDQDED EDEDEDQPRK SREWRSKKTQ PRRPRQEEPR ERGCETRNGV EENICTLKLH ENIARPSRAD FYNPKAGRIS TLNSLTLPAL RQFQLSAQYV VLYKNGIYSP HWNLNANSVI YVTRGQGKVR VVNCQGNAVF DGELRRGQLL VVPQNFVVAE QAGEQGFEYI VFKTHHNAVT SYLKDVFRAI PSEVLAHSYN LRQSQVSELK YEGNWGPLVN PESQQGSPRV KVA

Figure S7. Amino acid sequence of glycinin G4 (NCBI NP_001238008.1).

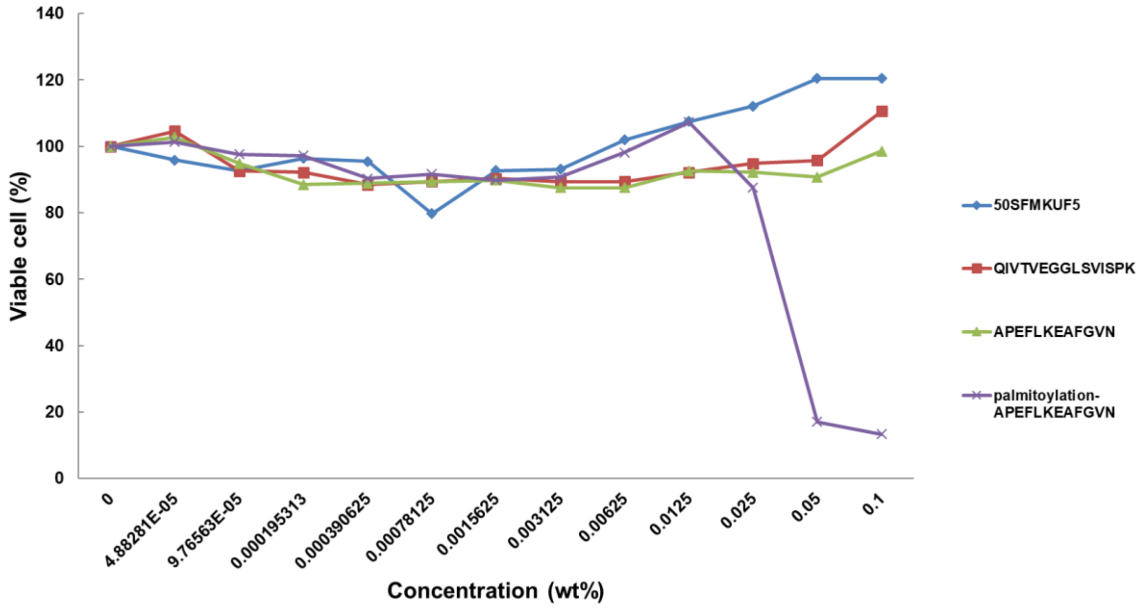

Figure S8. Effect of peptides on the viability of human dermal fibroblasts.

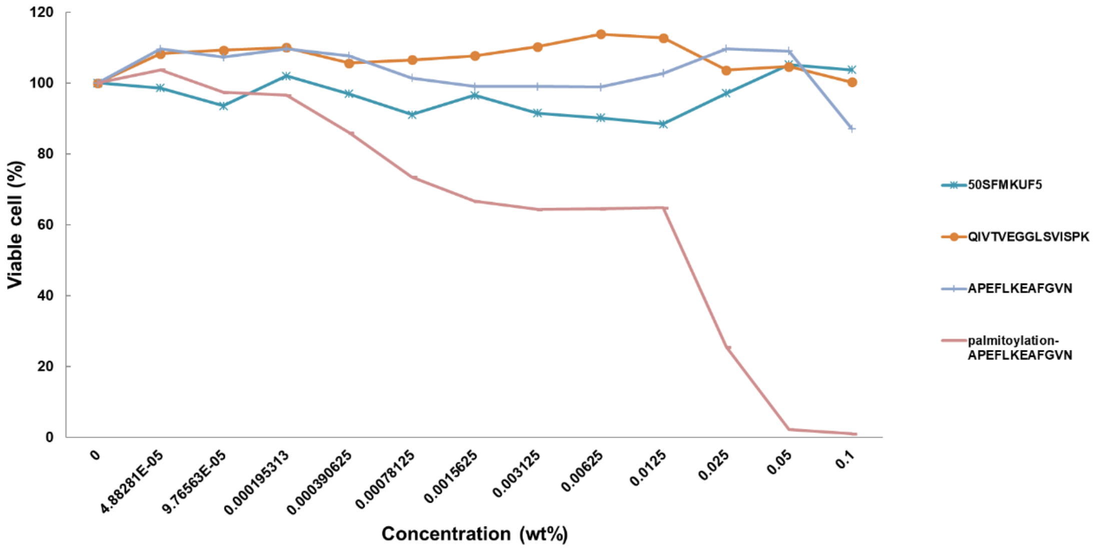

Figure S9. Effect of peptides on the cytotoxicity of HaCaT keratinocytes.

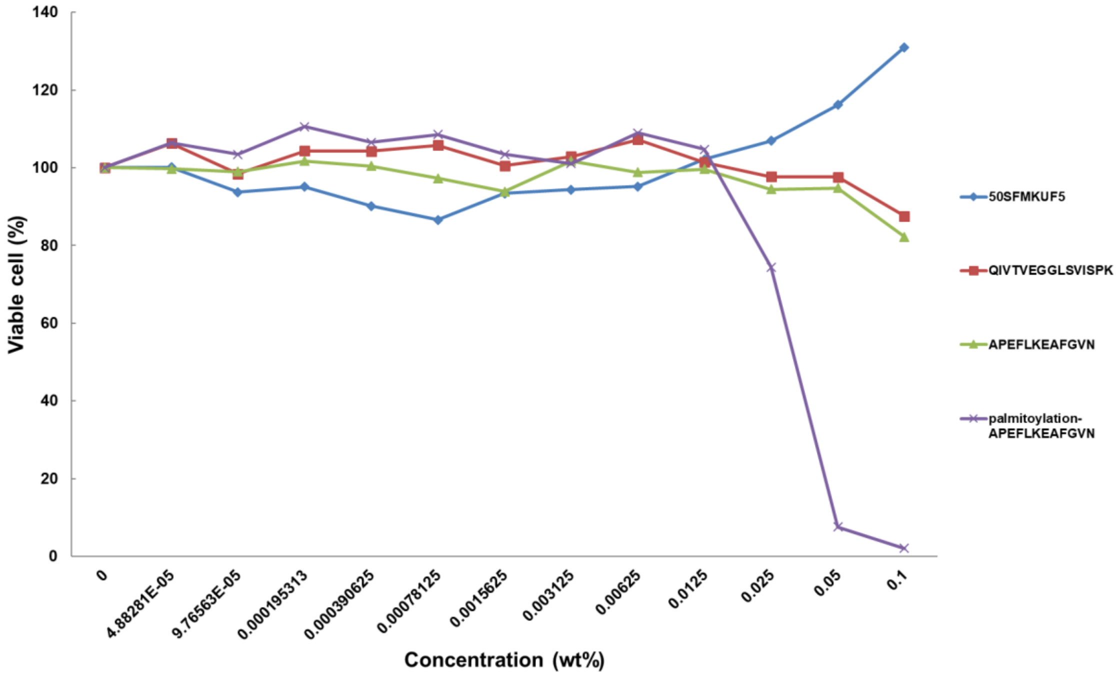

Figure S10. Effect of peptides on the cytotoxicity of B16F1 melanoma.
